# Evaluating Integration Strategies for Visuo-Haptic Object Recognition

**DOI:** 10.1007/s12559-017-9536-7

**Published:** 2017-12-28

**Authors:** Sibel Toprak, Nicolás Navarro-Guerrero, Stefan Wermter

**Affiliations:** Knowledge Technology, Department of Informatics, Universität Hamburg, Vogt-Kölln-Str. 30, 22527 Hamburg, Germany

**Keywords:** Object recognition, Multimodal integration, Brain-inspired architectures, Vision, Haptics, Tactile sensing

## Abstract

In computational systems for visuo-haptic object recognition, vision and haptics are often modeled as separate processes. But this is far from what really happens in the human brain, where cross- as well as multimodal interactions take place between the two sensory modalities. Generally, three main principles can be identified as underlying the processing of the visual and haptic object-related stimuli in the brain: (1) hierarchical processing, (2) the divergence of the processing onto substreams for object shape and material perception, and (3) the experience-driven self-organization of the integratory neural circuits. The question arises whether an object recognition system can benefit in terms of performance from adopting these brain-inspired processing principles for the integration of the visual and haptic inputs. To address this, we compare the integration strategy that incorporates all three principles to the two commonly used integration strategies in the literature. We collected data with a NAO robot enhanced with inexpensive contact microphones as tactile sensors. The results of our experiments involving every-day objects indicate that (1) the contact microphones are a good alternative to capturing tactile information and that (2) organizing the processing of the visual and haptic inputs hierarchically and in two pre-processing streams is helpful performance-wise. Nevertheless, further research is needed to effectively quantify the role of each identified principle by itself as well as in combination with others.

## Introduction

Most envisioned applications require robots to recognize objects well. Although vision is important for object recognition, there are certain hard-to-overcome challenges when relying on it alone [20]. Even the most advanced vision-based techniques are restricted in their performance by hardware limitations such as the quality of the camera, environmental conditions leading to poor lighting in the scene, and objects that are highly translucent or reflecting. Also, vision often cannot provide all the information needed about an object at once; what can be seen of an object depends on the viewpoint and on how much the object is occluded by other objects in the scene. Then, there is an inherent restriction to what exactly can be perceived with vision alone: There are certain objects whose identities can still be ambiguous even if the robot has a perfect view of them, a good example being decorative artificial fruits and vegetables that can sometimes look very much like real ones. The identity of an object is defined by its purpose and its purpose is defined by its material and form properties, which are not all visually perceivable.

In situations, where we cannot completely rely on our vision, be it due to the ambiguity of the object that we want to identify, external conditions or even visual impairments, we resort to other senses, most often to our “sense of touch.” With the additional sensory information obtained by manually exploring the objects, we are better able to recognize them. Robots can similarly benefit from additional haptic perception capabilities for the object recognition task, e.g., [25, 66]. Usually, the object recognition process is modeled in a way where the visual and haptic information extracted from an object are processed separately until they finally converge to a classification result. The human brain, however, deals with this information differently: interactions between vision and touch take place in the cortex [39]. These interactions can be crossmodal, meaning that the haptic stimuli activate regions traditionally believed to be visual, or multimodal, in which case the visual and the haptic stimuli converge. Incorporating these insights regarding how the human brain combines vision and haptics to recognize objects might help robots approach human proficiency, eventually. Therefore, the objectives of this paper are the following:

1. to present a functional description of how visuo-haptic object recognition is performed in the brain and derive organizational principles that could be used for robots (see “Visuo-Haptic Principles of the Human Brain”).
2. to review existing visuo-haptic integration strategies and assess to which extent these do (not) adopt the identified principles (see “Towards a Brain-Inspired Integration Strategy”).
3. to evaluate how using these principles in a unified brain-inspired integration strategy influences the object recognition performance in comparison with the commonly used integration strategies (see “Methods” and “Results”).

## Visuo-Haptic Principles of the Human Brain

The human brain’s proficiency in recognizing objects remains unmatched by existing artificial systems, which underlines the complexity of this task [34, 38]. Humans can identify objects instantly and accurately, even if only view-dependent partial observations are available caused by occlusion in cluttered environments or variances in the object’s pose. Moreover, our brain is highly robust to failures: If the information about an object acquired through vision is not reliable, it combines it with other sensory modalities.

An especially strong link exists between vision and haptics: If we see an interesting or unfamiliar object surface, we will intuitively feel the urge to touch and explore it. In many aspects, these two modalities are also quite complementary: While haptic perception is sequential in how the properties of an object are appreciated [41], visual perception is instantaneous in that multiple object properties can literally be perceived at one glance. Our skin, being our largest sensory organ, augments the limited perceptual space accessible with our eyes to cover more of our environment. Hence, a uniquely rich domain of observations is possible with the haptic system, especially when active touch is involved. The visually and haptically perceivable object properties constitute complementary sets for the most part as well.

Understanding the processes within the brain that are involved in the object recognition task, especially in making sense of the information coming from the different modalities, might help us building better computational systems with capabilities comparable to that of humans [34, 38]. Despite being a very actively researched topic, how exactly the human brain accomplishes object recognition is still not fully known. However, the following three processing principles can be identified as underlying the visuo-haptic integration in the cortex during object perception and recognition:

1. *Hierarchical processing:* Starting at the back of the brain and going ventrally along the cortex, the object-related visual stimuli is processed hierarchically and abstracted into higher-level features (see “Hierarchical Processing Along the Ventral Pathway for Object Identification”).
2. *Parallel processing of shape and material properties:* The processing disperses into two streams, one dedicated to the perception of shape and the other to the perception of the material properties (see “Substreams for Object Shape and Material Processing”), and involves haptic activations in both of these streams (see “Haptic Activation Along the Substreams”).
3. *Experience-driven self-organization:* The ability to process and integrate the information from the different sensory modalities optimally is not hard-coded in the brain but develops after birth (see “Input-Driven Self-Organization of the Underlying Neural Circuits During Visuo-Haptic Integration”).

### Hierarchical Processing Along the Ventral Pathway for Object Identification

Every sensory system that a human possesses is associated with an area of the cortex that is its primary sensory area. This is the earliest cortical area to process the stimuli coming from the receptors of that particular sensory system. For the visual sense, the primary sensory area is the primary visual cortex (V1) [28, 38] located in the occipital lobe. The orderly arrangement of the retinal cells’ receptive fields is preserved in how the visual stimuli are projected onto V1; this particular case of topographic organization is called retinotopy. The visual features processed in this earliest area are rather simple and low-level. The output of the processing is forwarded to the subsequent visual cortical areas. These areas are organized hierarchically so that the low-level inputs are successively transformed into more complex and abstract representations [28, 38, 64]: As one proceeds from one area to the next, the receptive fields of the cells get larger and the complexity of the features they respond to increases.

**Fig. 1:**
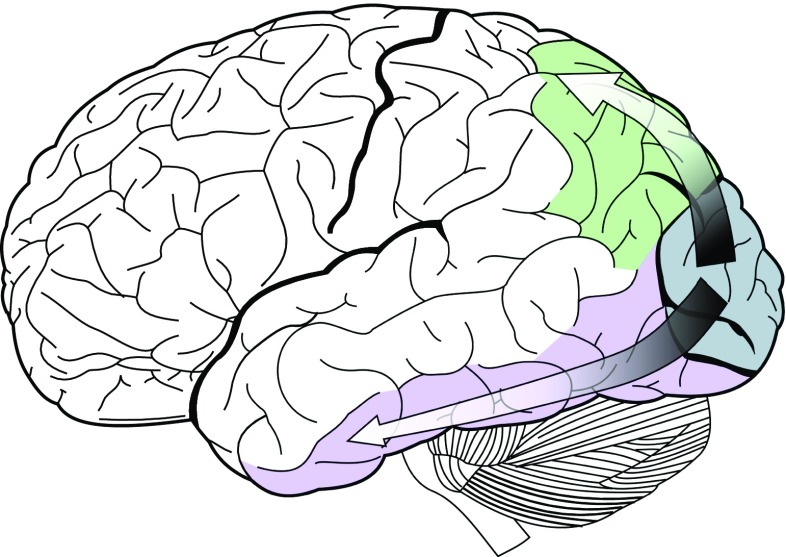
The dorsal () and ventral () processing streams emanating from V1 (). (*Image source: Wikipedia*)

It is widely accepted that the further processing of the visual stimuli diverges onto two functionally separate streams starting from V1 [46, 64], as shown in Fig. 1. One stream extends to the posterior parietal cortex. This dorsal processing stream is sometimes also referred to as the “where pathway” or “how pathway.” It is said to be in charge of perceiving the spatial relationships among objects and coordinating visually-guided actions directed at objects, such as reaching and grasping. The other stream reaches as far as the inferior temporal cortex. This ventral processing stream is also known as the “what pathway” which is in charge of recognizing objects. Hence, this is the stream that is of interest in the context of this work. The two streams have also been described alternatively as “action” and “perception” pathways [26].

### Substreams for Object Shape and Material Processing

There is significant evidence that the ventral stream is functionally further specialized into separate processing pathways, for the object form and surface properties respectively [13, 14]: The posterior-lateral regions of the occipitotemporal cortex, encompassing the lateral occipital area (LO), were shown to be engaged in object form perception. The perception of surface properties such as texture and color, on the other hand, is dealt with in more medial areas of the ventral pathway. Here, the area along the collateral sulcus (CoS) was identified as texture-specific, but no color-specific area was found. It is believed that the extraction of information about surface color occurs relatively early in the ventral stream compared to surface texture. Interestingly, there seems to be an overlap between regions that are form-selective and those thought to be involved in object recognition. The same appears to be the case for regions that are selective to surface properties and those that have been associated with face and scene recognition.

These findings were confirmed and extended in further studies [16, 17]: The medial portion of the ventral pathway was found to be organized into multiple “foci,” where each one processes the stimuli related to a particular surface property. These interact with each other to perceive the material properties of an object. A texture-selective region appears to be located posterior to a color-selective one. Also, regions responsive to shape, texture and color together were found to be located beside regions exhibiting single-feature selectivity [16, 17]. These areas lie within the fusiform gyrus (FG) in the temporal lobe, apparently corresponding to the same areas associated with the perception of more complex stimuli such as faces and places, which is in line with existing evidence [14].

### Haptic Activation Along the Substreams

The primary sensory area for the sense of touch is the primary somatic sensory cortex (S1) [34, 56] located in the postcentral gyrus of the parietal lobe. It processes the sensory inputs that it receives from the tactile and kinesthetic senses, typically during active manual exploration [19], for the purpose of texture and form discrimination. Whereas the tactile stimuli come from receptors distributed over the whole skin surface, the kinesthetic stimuli come from receptors that are embedded in the muscles, joints and tendons [19]. As in V1, these stimuli are also processed in a hierarchical manner here. S1 is organized somatotopically, a special case of topographical organization like retinotopy, meaning that there are complete maps of the body at different levels of processing. The body is not represented in actual proportion; the area dedicated to each body part in S1 directly reflects the density of receptors contained in it.

**Fig. 2:**
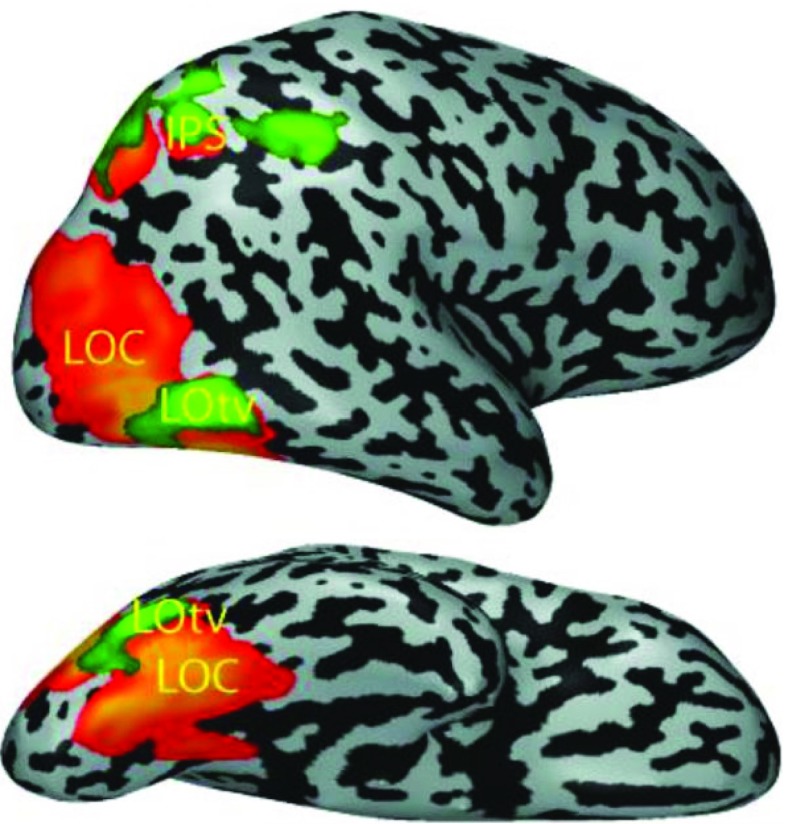
Regions in the LOC exhibiting visual () and haptic () object selectivity shown on the right brain hemisphere (top: lateral view; bottom: ventral view). (*Image source:* [40], with permission of Springer.)

The functional divergence onto separate pathways might not be specific to the visual system alone. It is very likely that there is an analogue in the somatosensory system with two or potentially more separate pathways [33, 58]. However, different views exist regarding how the neural substrates underlying haptic perception are organized into such streams (see [33] for a review of three of them). Object-related haptic activation outside the somatic sensory cortex has been found in certain regions along the ventral visual pathway. We already know the lateral occipital complex (LOC) for its involvement in visual shape processing [37, 43]. Figure 2 shows the part of the LOC that responds selectively to objects in both vision and haptics. This subregion has been named lateral occipital tactile-visual region (LOtv). Based on this finding, it was concluded that the LOC is a bimodal convergence area involved in the recovery of the geometric shape of objects [4, 5]. That the neurons in the LOtv are truly bimodal and form a visuo-haptic object-related integration site was confirmed later [61]. Haptic activation related to the perception of surface texture also occurs in certain regions within the medial occipitotemporal cortex [54, 55, 65]. These regions are not bimodal in the same way that the shape processing regions are the following: While they are in very close proximity to those on the CoS responsive to surface texture during visual perception, they are spatially distinguishable. It may be that the visual and haptic texture information are represented differently from the visual and haptic shape information. However, it is possible that the processing is not completely independent and that the regional adjacency facilitates crossmodal interaction.

Interestingly, there is evidence that object weight is represented in the ventral visual pathway as well, specifically in the medial regions [24]. This might explain what we know from our own experience: We can directly perceive the weight of an object when we explore it haptically but we can also infer this information from the visually perceived properties. Also, an object’s weight is a part of its identity, as it contributes to its distinguishability from other objects. Hence, this finding is indeed in line with the ones presented above, especially the ones related to the role of the ventral visual pathway in object recognition [35]. It also gives rise to the assumption that other properties, such as object hardness, are dealt with in a similar way.

### Input-Driven Self-Organization of the Underlying Neural Circuits During Visuo-Haptic Integration

Our brain integrates object-related visual and haptic information in a manner that is statistically optimal, weighting each sense according to its reliability [21,30]. Not much is known about the particulars of how the neural substrates of object perception combine the visual and haptic experiences to more abstract and meaningful perceptual experiences for learning or recognition. There are many indications though that this capability, in general, is not an inherent one. It is a result of *self-organization* among the neurons driven by the experience accumulated during development, that is, *input-driven* [45].

Multisensory integration at the level of single neurons has been studied primarily in the cat superior colliculus (see [60] for an extensive review). Newborn cats are already capable of detecting certain cross-modal correspondences but not of integrating the information coming from the different senses. The latter develops after birth based on their cross-modal experiences of the environment, to which the underlying neural circuitry adapts in a way that optimizes their multisensory integration capabilities. The contributing unisensory systems do not have to reach a final point in maturation for this development to begin; both happen in parallel.

The occurrence of self-organization in the primary sensory areas suggests that it is a fundamental mechanism in the brain. The V1 neurons respond selectively to certain features such as orientation and color, forming different cortical feature maps. There is a pattern to how these feature preferences are spatially organized in these maps [45]: Their coarse structure, which is fixed before birth, is determined by retinotopy, whereas the finer structure emerges only after birth, being shaped by visual experience. Experiments have shown that physiological deficits of varying degree can be caused in kittens by depriving them of normal visual experience, especially in the first few weeks after birth, a critical period in their development: Suturing their eyelids shut causes disorganization in the visual cortex and can even lead to permanent blindness if the eyes are kept that way until after the critical period (e.g., [32]). Kittens exposed to an environment consisting entirely of horizontal or vertical stripes for some time have a lot of difficulty processing complex visual scenes (e.g., [9,10]). The somatic sensory maps develop in a dynamic fashion as well, possibly starting as soon as the first body movements occur in the womb [47].

The most convincing argument in favor of our visuo-haptic integration capabilities being the result of input-driven self-organization comes from a behavioral study with humans [27]. It seems that a human’s ability to integrate visual and haptic information during object form perception becomes statistically optimal only at the age of 8 to 10. Until then, the importance that children give to either modality often does not reflect its reliability in a particular situation. That being the case, there must be some “trigger” that causes associative links between unimodal experiences to be established. Perceptual illusions, such as the *rubber hand illusion* (RHI), reveal that the temporal co-occurrence of visual and haptic stimuli is important. There is also a spatial aspect to it: The integration of the two modalities becomes likelier if it is known that the stimuli come from the same object [30].

## Towards a Brain-Inspired Integration Strategy

The review in “Visuo-Haptic Principles of the Human Brain” of how the human brain performs visuo-haptic object perception has revealed that the processing of the visual and haptic information is organized hierarchically, that it diverges onto two separate processing streams, one dedicated to object shape perception and the other to object material perception, and that the mechanisms behind the processing and integration of information are self-organized. In this section, we review existing integration strategies and we describe up to what extent the identified brain-inspired processing principles have been tried already.

### Existing Work on Visuo-Haptic Object Recognition

The idea of integrating vision and touch for the purpose of generating descriptions of object surfaces dates back to 1984 [2] and has been extended to encompass the whole object recognition task [3]. Since then, a lot of work has been done on recognizing objects based on either vision or haptics alone [66]. Despite the significant progress achieved in object recognition based on either modality, the combination of both has attracted less attention in comparison [66]. The relevant approaches are listed in Table 1. There, they are roughly organized into three main categories according to the taxonomy of information fusion approaches presented by Sanderson and Paliwal [57], namely *pre-mapping*, *midst-mapping* and *post-mapping fusion*. The difference between these approaches is the point at which the information coming from the different sources (here modalities) converges in the process of mapping from the feature space to the decision space.

**Table 1:** Existing work on visuo-haptic object recognition organized according to Sanderson and Paliwal’s taxonomy of information fusion approaches [57]

Related work		Information fusion approaches		
		Pre-mapping	Midst-mapping	Post-mapping
Güler et al. (2014)	[25]	×		
Yang et al. (2015)	[66]		×	
Liu et al. (2017)	[42]		×	
Nakamura et al. (2007)	[49]		×	
Corradi et al. (2017)	[18]	×	×	
Castellini et al. (2011)	[15]	×	×	×

#### **Pre-mapping Fusion**

In pre-mapping fusion, all available feature descriptors are simply concatenated into a single vector before the mapping on the decision space is performed. Güler et al. [25], for instance, use this approach in their work on identifying the content inside a container based on vision and touch: A three-fingered Schunk Dextrous Hand explores a cardboard container, either empty or filled with water, yoghurt, flour, or rice, by performing grasping and squeezing actions on it. The effects of these actions on the container, that is, the pressure exerted on the contact points and the deformation achieved, are extracted as touch and visual cues, respectively. The visual data is collected with a Kinect camera that is placed one meter away from the robot platform and consists of the change in depth values around the contact points registered during the grasp. The tactile data comes from the touch sensing arrays on the fingertips of the robot hand. After standardizing the data to zero mean and unit variance, principal component analysis (PCA) is applied to the visual and tactile data combined to cope with the high dimensionality. The combined data then serves as input to different supervised and unsupervised learning methods, namely k-means, quadratic discriminant analysis (QDA), k-nearest neighbors (kNN) and support vector machines (SVM). The results show that the classification accuracy benefits from combining visual and haptic input. However, there is one general problem with the pre-mapping fusion approach in that there is no explicit control over how much each modality contributes towards the final decision [57]. The influence of each modality is proportional to how much its respective feature vector makes up the combined feature descriptor.

#### **Midst-Mapping Fusion**

In midst-mapping fusion, the feature descriptors are provided to the system separately, which then processes them in separate streams and integrates them while performing the mapping.

An example of this information fusion approach is the method that Yang et al. [66] propose for visuo-tactile object recognition of common household objects. The visual information is extracted from an image of the object by means of the so-called covariance descriptor (CovD). This representation is obtained by computing the covariance matrix for a set of feature vectors, where each vector is associated with one pixel. Meanwhile, the tactile input is composed of the tactile sequences coming from the sensors of the three-fingered BarrettHand^TM^. A kNN classifier finally performs the fusion of these representations. The weighted sum of distance measures defined for each sensory modality is used as the overall distance measure for determining the nearest neighbors. Depending on how the weight values are chosen, the kNN classifier’s input can range from tactile-only to visual-only information. These results support the conclusion that the performance of the combination is better than relying on a single modality [25]. Here, the authors experiment with different weightings of the modalities, but the downside is that the weights have to be set manually by trial and error.

Liu et al. [42] employ the same hardware and feature extraction techniques but a different visuo-tactile integration method, which is based on kernel sparse coding (KSP). KSP maps data to a high-dimensional feature space and generates a dictionary of atoms in terms of which the data can then be encoded sparsely. However, this method fails to capture the intrinsic relations between the different data sources: It can only be applied to each modality separately, resulting in two different coding vectors. The authors address this problem by proposing an extension, which they call kernel group sparse coding (JKGSC). By taking into account constraints that characterize the intrinsic relations between the different modalities, JKGSC produces for each modality coding vectors that are pattern-wise similar to each other for the same object. The results of their experiments show that fusing the visual and tactile information using the JKGSC method leads to a higher classification accuracy than applying the KSP method to each modality separately.

Another example of the midst-mapping fusion approach [48,49] endows a robot with the capability of exploring objects and categorizing them in an unsupervised fashion based on the gathered visual, haptic and auditory information. The robot grasps an object, inspects it from different viewpoints and listens to the sound it makes. The data collected in the process comprises the images taken with the robot’s camera and the signals recorded with the microphones and the pressure sensors on the robot’s hand. The information is represented in a bag-of-words (BoW) model for each modality. A codebook is generated by identifying patterns of co-occurring features, referred to as codewords, using k-means clustering over the detected features. The codewords are then the centers of the learned clusters. The visual, haptic and auditory information collected about a single object over the entire observation period is described in terms of the respective codebook by counting the frequency of occurrence of each codeword in that information. The number of codewords in a codebook determines the length of the final representation. The codebook for the visual modality is generated from descriptors computed with the scale-invariant feature transform (SIFT) algorithm for the salient regions in the image data and contains 600 codewords. The haptic information is comprised of the sum of the digitized voltages coming from the pressure sensors and the change in angle between the two fingers until the completion of grasp, resulting in a codebook with five words for this modality. Finally, a codebook of size 50 is obtained for the auditory modality from the Mel-Frequency Cepstral Coefficients (MFCCs) computed for the speech and noise signals. The categorization of the objects is carried out with a proposed extension to the probabilistic latent semantic analysis (pLSA). The evaluation results show that the proposed method performs best when all three modalities are included. An advantage is that it learns the optimal weighting of the modalities.

Corradi et al. [18] perform a comparison between two midst-mapping fusion approaches and one pre-mapping fusion approach. A KUKA KR-650 arm is used to explore objects. The tactile sensor mounted on this arm consists of an enclosure covered by a silicone rubber membrane, with a camera and several LEDs, illuminating the membrane, inside. The rubber deforms when in contact with an object and creates shading patterns that are then captured by the camera. After normalizing the camera output, Zernike moments are computed and PCA is applied to reduce the dimensionality to 20. As for the visual data, the BoW model is applied to the speeded up robust features (SURF) extracted from it to create a codebook of size 100. The visual and tactile information are then fused in three different ways: (1) The extracted feature vectors are concatenated and the object label is predicted with kNN, (2) the posterior probabilities (the probability of the label given the observation) are estimated for each modality and the object label that maximizes their product is chosen, and (3) the object label that maximizes the sum of these posterior probabilities weighted by the number of training samples available for each modality is chosen. All three fusion approaches yield higher classification accuracies than either modality alone. When compared with each other, the posterior product approach outperforms the other two.

#### **Post-Mapping Fusion**

In post-mapping fusion, the mapping from the feature space to the decision space is performed on each feature separately before the resulting decisions are finally combined. Castellini et al. use this approach in their work on recognizing everyday objects using visual and kinesthetic input [15]. Actually, they test all three information fusion approaches and compare the results. The data comes from recording the act of grasping an object in a particular way performed by different subjects on different objects. From the obtained image sequences, some frames are selected in which the object is perfectly visible. The regions of interest (ROIs) showing the object are identified by performing background subtraction and change detection with respect to a background model on the remaining frames. The SIFT descriptors extracted from these ROIs are used to construct the BoW model resulting in a codebook with 200 codewords. The kinesthetic information is captured with the CyberGlove, a sensorized glove. The posture of the hand when grasping the object is described with a total of 22 values. The proposed classifier handles two conditions: In one condition, both visual and haptic object-related information are available, while in the other, only the visual portion is available. In the latter case, the motor features are reconstructed from the visual information. The classifier is implemented in an SVM-based framework and can combine the visual and haptic cues on three different levels. Again, the conclusion is that the integration of multiple modalities improves the classification performance. Among all three approaches for information fusion, the mid-level one achieves the highest accuracy, closely followed by the high-level approach.

### Relating the Existing Work to the Identified Brain-Inspired Processing Principles

The review of the existing work reveals a concentrated effort to prove the benefits of integrating vision and haptics for the object recognition task. Although the reviewed research covers a number of different integrations strategies, they all lack a clear rationale for why a particular integration strategy was chosen. To the best of our knowledge, a comprehensive comparison has only been attempted by Castellini and colleagues [15] thus far. Their results suggest that midst-mapping fusion is the most promising approach in terms of performance. At the same time, it is the information fusion approach that has been pursued the most often according to Table 1 and that also reflects by definition two of the processing principles seen in “Visuo-Haptic Principles of the Human Brain”: The processing of the visual and haptic inputs is organized hierarchically and in two processing streams.

What has not been examined yet is organizing the streams to process object shape and material separately (cf. the second principle, see “Substreams for Object Shape and Material Processing” and “Haptic Activation Along the Substreams”). Interestingly, in all instances of midst-mapping fusion presented above, the processing of the information is separated based on the modality instead. One reason for this rather straightforward organization might be that the small number of object properties that are considered does not allow for a more sophisticated combination of the visual and haptic inputs. Although there are many more haptically perceivable object properties, for example, the only ones that have been taken into account in the presented works are the object’s shape or hardness. Another reason might be the use of global general-purpose feature descriptors, like SIFT, instead of decomposing the information into discrete object properties. Also, none of the presented research makes use of self-organizing mechanisms for the processing and integration of the visual and haptic object-related information (cf. the third principle, see “Input-Driven Self-Organization of the Underlying Neural Circuits During Visuo-Haptic Integration”).

**Fig. 3:**
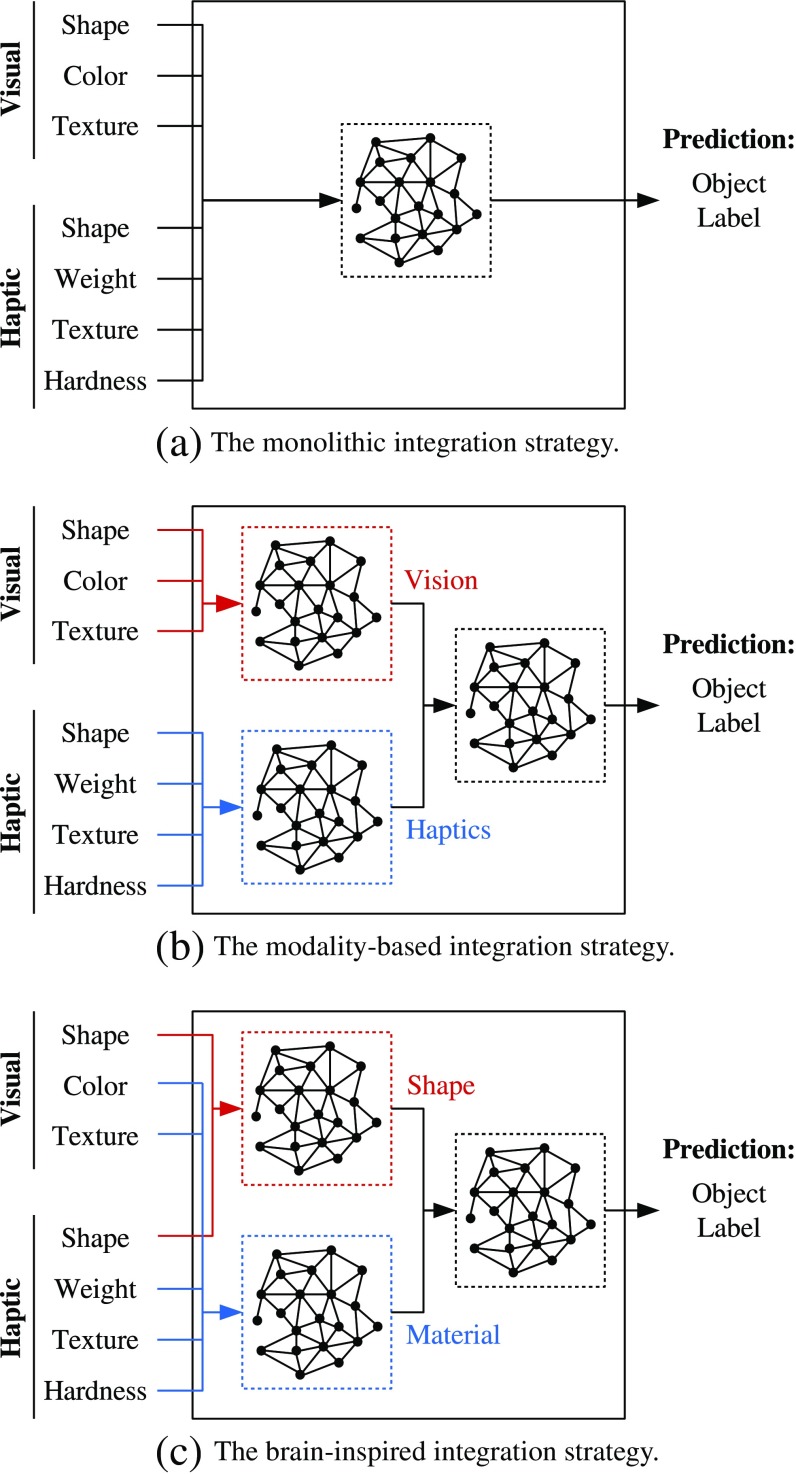
Integration strategies

The question arises whether pursuing the brain-inspired processing principles for object recognition outlined in “Visuo-Haptic Principles of the Human Brain” would be beneficial for robot applications. To explore this question, we examine the three integration strategies illustrated in Fig. 3: (a) The *monolithic* integration strategy, which is an instance of the pre-mapping fusion approach and therefore performs the classification on the concatenation of all object descriptors, (b) the *modality-based* integration strategy, which is used the most often and where the visual and haptic inputs are processed in two separate streams before the results of the pre-processing are integrated in the final object label predictor, and finally (c) the *brain-inspired* integration strategy, which incorporates all of the above-mentioned processing principles. It is also an instance of the midst-fusion approach, but unlike in the previous integration strategy, the shape- and the material-related inputs are pre-processed separately so that the integration of the modalities takes place as soon as the first layer.

## Methods

This section presents the various methods used for collecting data, extracting the relevant visual and haptic features and implementing the neural classifiers that combine the information from the two modalities according to the three integration strategies to be compared.

### Data Collection

#### **Robot Platform**

The object exploration was performed with the humanoid robot NAO by SoftBank Robotics. It is available in different body types; we used the torso-only model, NAO T14. One advantage of using the NAO robot is that it offers sensing capabilities for most of the object properties that we wish to extract: The visual data can be collected with either one of the two RGB cameras built into its head. For the haptic data for the object properties that involve kinesthesis, namely shape and weight, the joint angle values and the currents delivered to the motors in both arms can be used. Both types of information are readily accessible via the standard API (v2.1.4.13).

On the other side, NAO lacks sensors for the other two haptic object properties, which are related to tactile perception, namely haptic texture and hardness. While here are capacitative tactile sensors on some parts of the body including the head and hands, these are meant for user input. Our solution to this problem is inspired by Harrison and Hudson [29]: Here, an inexpensive sensor was incorporated into a mobile device to capture user input in the form of sounds produced with a finger on the surface below the device. Similarly, such inexpensive sensors could be attached to NAO’s arms or the table below to record the vibrations transmitted across the surface during object exploration. The robot can then perform simple scratching and tapping actions on the object to explore its texture and hardness, respectively. Ideally, the sensors could be placed on the skin surface from inside the robot arm. We use contact microphones as sensors. When in contact with solid objects, this form of microphone is very sensitive to structure-borne vibrations, but pretty much insensitive to air-borne ones. The use of microphones is also biologically plausible: It allows to emulate how the so-called *mechanoreceptors* work, which are one type of somatic sensory receptors that react to mechanical pressure or vibration [56].

In particular, we use the inexpensive Harley Benton CM-1000 clip-on contact microphones and the 4-Channel ALESIS iO4 audio recording interface in order to be able to work with multiple sensors at the same time. Two of the contact microphones are attached to the table, one clipped onto the end of the ridged stick pointing towards the robot and the other clipped onto the edge of the table surface on the opposite side. The other two microphones are attached to the left arm of the robot, see Fig. 5. To make things short and easy, the contact microphones will henceforth be referred to as channels 0 to 3, based on the number of the input channel they were assigned to on the audio interface, in the order mentioned.

#### **Object Exploration**

NAO’s three-fingered hands only allow for very small and light objects to be held in one hand. The problem is that the fingers then completely cover the objects so that no surface area is left for the other hand to explore haptically. Bigger objects are much more likely to vary in weight, which makes the use of this property to distinguish between the objects meaningful, but require the robot to hold them with both hands. Having it hold the object throughout the whole haptic exploration process means that there will be no free hand to explore the object’s texture and hardness. Hence, a custom table was built for the robot to explore objects on, with a stick to serve as an additional finger attached on top. Small ridges were added to this stick, inspired by the fine ridges on our fingertips, to increase the friction when in contact with an object surface. The overall sequence of exploratory movements that the robot performs was hard-coded. For a smooth execution, some human intervention is necessary at certain points in time to make sure that the object is in the expected spot.

The overall object exploration process is organized in two phases: In the first phase, visual-only exploration takes place. The robot opens up its arms to move them out of the way for a perfect view and waits for the object to be placed within its visual field. Once the visual data is collected, the object is positioned on the table so that, upon moving gradually closer to each other, the hands end up enclosing it. Once the robot has grasped the object, the second phase starts and the object is explored haptically. After having appreciated the shape of the object in its grasp, the robot lifts it up to weight it and then places it back down on the table surface. Then the robot moves the object laterally along the ridged stick on the custom table and hits it against the same stick three times to perceive its surface texture and hardness, respectively. Finally, the robot releases the object.

#### **Object Set**

**Fig. 4:**
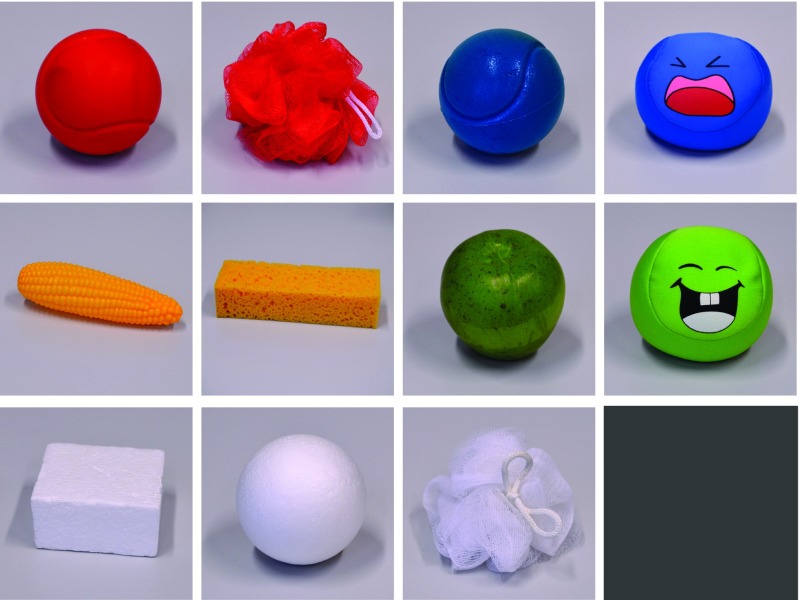
The set of objects used for the data collection from left to right, [top to bottom: red softball, red mesh sponge, blue softball, blue footbag, toy corn, sponge, decorative apple, green footbag, styrofoam piece, styrofoam ball], white mesh sponge

The set of 11 objects used in the data collection is shown in Fig. 4. It is comprised of objects big enough to not slip through the robot’s hands or be completely covered by the fingers. At the same time, they are small and light enough for the robot to pick them up with both hands without the fingers being in danger of being damaged. The choice also covers both visually and haptically ambiguous objects. Examples of objects that are visually ambiguous are the blue softball and the blue footbag, which are very similar in their visible shape, texture and color. The red and blue softballs are examples of the latter, the only difference being their colors. Not included are translucent objects, although they would have been quite interesting, in particular, because they pose a challenge to any object recognition techniques that are based on vision only.

#### **Conditions**

**Fig. 5:**
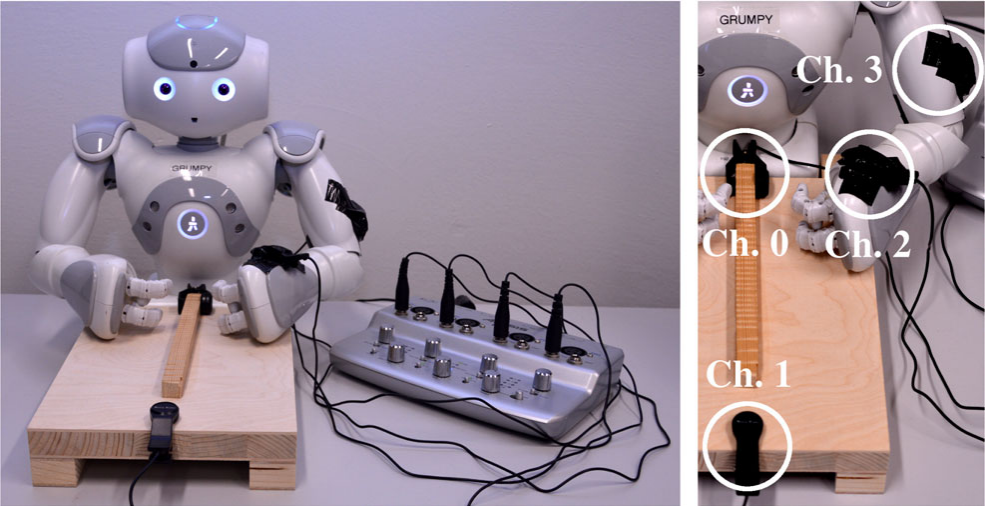
Left: The overall robotic set-up for the data collection, Right: The placements of the four contact microphones (two of them on the table and the other two on the robot’s arm)

The overall data collection set-up is shown in Fig. 5. It consists of the robot, a custom table that it explores the objects on, the four clip-on contact microphones and the audio interface that all of them are connected to.

The data was collected under two conditions. First, ten observations were collected for every object in the object set under controlled and reproducible lab conditions: The ceiling lamps were switched on and the curtains were closed. A standing lamp was placed behind the robot to light the surface of the custom table and to ensure that the objects placed on top during visual exploration cast as little shadow as possible, which yields better object segmentation results. The observations for each object were added in a 70:30 ratio to the training and test sets. Another three observations per object were collected under uncontrolled real-world conditions: The standing lamp was removed from the set-up and the curtains were opened. We let the robot perform some of the object exploration runs in a slightly imprecise manner. The assignment of these additional observations to the training and test sets was done in the same way as above.

### Feature Extraction

From the collected image, sound and motor data, the feature descriptors for the objects’ visual and haptic shape and surface texture, color, weight and hardness were extracted.^1^

#### **Visual Features**

Images were taken with NAO’s lower head camera with a resolution of 1280 × 960 pixels, which were then processed using standard image processing techniques from the open-source Python libraries OpenCV (v3.1.0) [12] and scikit-image (v0.17.1) [53]. Two images were collected in each object exploration run, one of only the scene background and the other of the same scene with the object in it. *Background subtraction* [59] was applied to each such pair of images to segment the objects before finally extracting the shape, color, and texture descriptors from the identified regions of interest.

The shape descriptor is composed of the seven so-called *Hu moments* [12,31]. These result from different linear combinations of centralized and normalized moments. Usually, the first and seventh Hu moments have the largest and smallest values, respectively. The color descriptor is obtained by concatenating the normalized color histograms for each color channel in BGR order, resulting in a total length of 768. As for the texture descriptor, it is a normalized histogram of so-called *local binary patterns* (LBP) [50] with 26 entries in total. The LBPs capture textural patterns (primitive micro-features, such as edges, corners and spots) defined by local pixel neighborhoods: The differences in gray value between a center pixel and its neighboring pixels are encoded as a binary pattern which is then converted into a decimal representation.

#### **Haptic Features**

The four haptic object properties that are considered here can be divided into two groups: One group is comprised of shape and weight, which are kinesthetically perceived object properties, while the other contains the tactile object properties texture and hardness. The data for each group was collected from a different source.

The kinesthetic data is comprised of the joint angles and electric currents measured in both of the robot’s arms. The joint position of the arms retrieved after the robot has fully enclosed an object with both hands is used as the shape descriptor. With 6 degrees of freedom (DOF) in one arm, its overall length is 12. As for the weight descriptor, the electric currents recorded in both arms while the robot is holding up the object were used in addition. The assumption is the following: The more an object weighs, the higher the electric currents will be in order to compensate for gravity and to reach the desired joint position with the arms. With the joint positions (both desired and actually achieved) and the electric currents, this descriptor is comprised of 36 values.

**Fig. 6:**
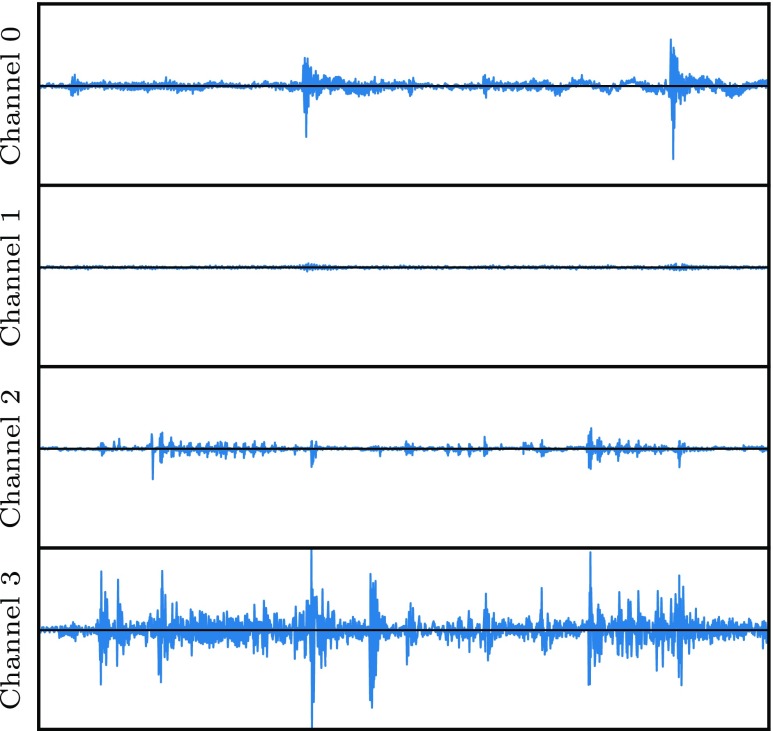
An example of the clean signals (one for each channel) obtained after performing spectral subtraction on the 1 s sound snippets that were recorded during the haptic texture exploration of the red ball

The tactile data was collected with the clip-on contact microphones attached to different places of the overall data collection set-up. One-second sound snippets were recorded with a sampling rate of 44,100 Hz before and while the robot executed the exploratory movements for texture or hardness. On each pair of sound snippets, *spectral subtraction* [11] was performed to suppress the background noise and thereby isolate the tactile vibrations. Figure 6 shows one example of the resulting clean signal. The final descriptor is the one-sided magnitude spectrum of the resulting Fourier transform, which is computed by taking its absolute value. It has 22,050 dimensions in total.

### Data Preparation

When all the feature descriptors are taken together, the number of dimensions amounts to 44,949. The problem is that training a classifier on such high-dimensional data requires a lot of time. For that reason, *principal component analysis* (PCA) [62], which is a dimensionality reduction technique that projects data onto a lower-dimensional space while keeping the loss of variance at a minimum, was performed separately on each portion of the data corresponding to an object property. The transformation matrix was computed for the training set data and then applied to the data in the test set. Multiple runs were performed in order to identify the number of dimensions that the variance in the original data can be fully explained with. In addition, all columns in the resulting data were *standardized* [62] to zero mean and unit standard deviation to prevent that those with larger values have the largest impact on the classification results.

### Classification

To incorporate the third processing principle identified in “Visuo-Haptic Principles of the Human Brain”, the integration strategies shown in Fig. 3 were implemented on the basis of a self-organizing neural network model, namely the *growing when required* (GWR) [44]. Unlike the popular *self-organizing map* (SOM) [36], it has the advantage of not being limited in its learning capability by being fixed in its size and topology. It is capable of growing like the *growing neural gas* (GNG) [22,23], without the drawback that a new node can only be added every time a certain number of adaptation steps have passed. The GWR can grow whenever necessary, can adapt better to any changes in the input distribution and converges faster. These characteristics make the GWR ideal to model the ongoing adaptive behavior of the brain cortex after birth.

#### **Training Algorithm**

The algorithm for training a GWR is outlined in Algorithm 1. Basically, the neurons of the network compete for the ownership of every input vector presented to it. The decision of whether a new node is necessary is then made based on the activity and the habituation of the best matching node. If its activity in response to the input vector is not high enough despite having been trained enough times, a new node is needed to better represent the input vector. This node is inserted into the network between the best and second best matching nodes. If the best matching node is not sufficiently trained yet, the best matching node and its neighboring nodes are moved closer to the input vector instead, the latter to a lesser extent. 
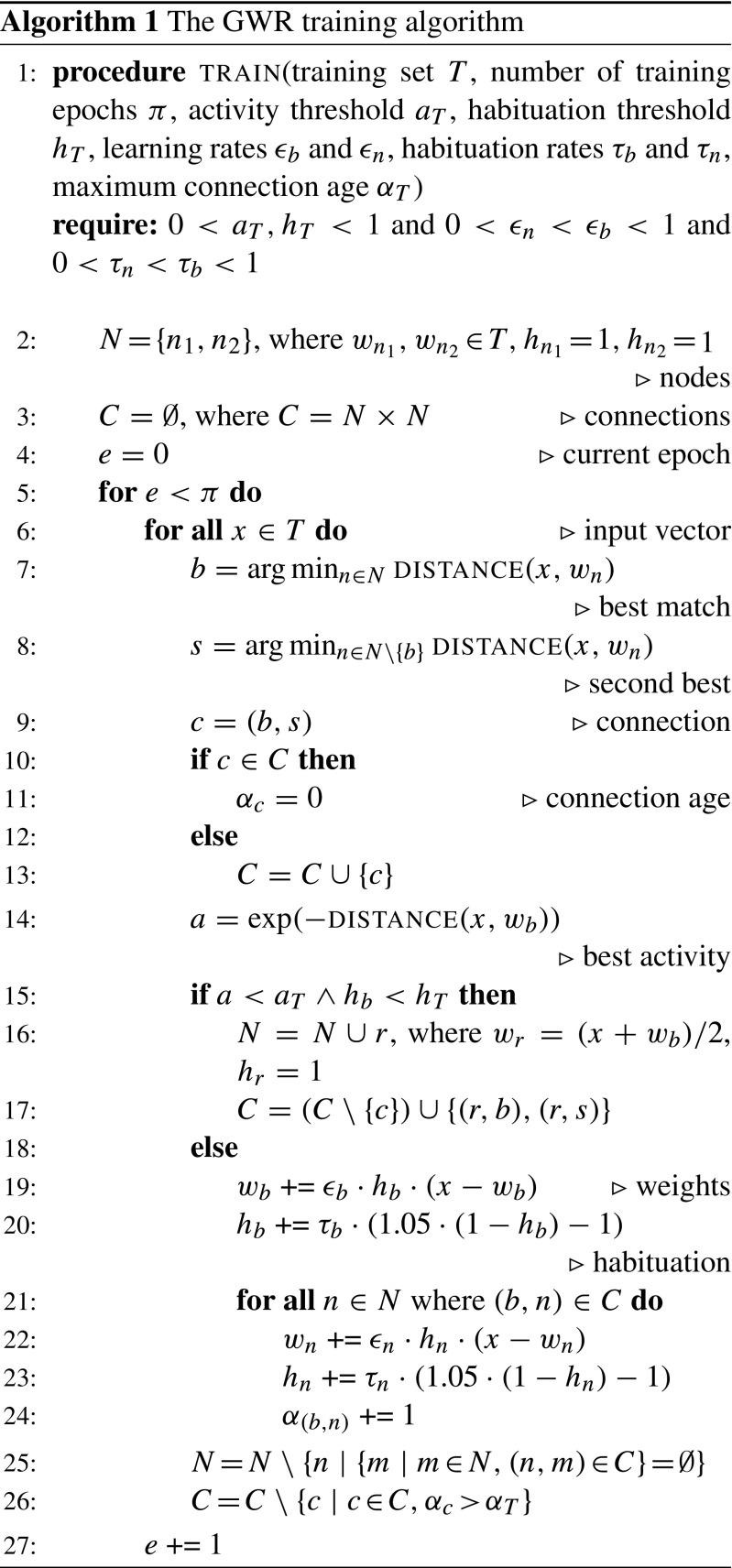


The following hyperparameters are required for the training process: The number of epochs *π* indicates how many times the GWR network is exposed to the entire training set. The activity threshold *a*_*T*_ specifies when the activity of the best matching node is considered high enough, while the habituation threshold *h*_*T*_ specifies when the node is trained sufficiently. Both thresholds are between 0 and 1. The two learning rates *𝜖*_*b*_ and *𝜖*_*n*_ control to what extent the weight vectors of the best matching node and its neighbors are adapted. The values should be chosen so that 0 < *𝜖*_*n*_ < *𝜖*_*b*_ < 1 holds. There are two habituation rates *τ*_*b*_ and *τ*_*n*_ as well. Apart from controlling how fast the habituation of the best matching node and its neighbors should converge to zero, these rates play a role during weight adaptation. Similar to before, the values should be chosen so that 0 < *τ*_*n*_ < *τ*_*b*_ < 1 holds. *α*_*T*_ is the maximum allowed age before a connection is removed from the GWR network. Finally, although actually not a hyperparameter, the distance function Distance is treated as one here.

#### **Implementing the Integration Strategies**

Implementing the three integration strategies in Fig. 3 using GWR networks as building blocks involves figuring out how to integrate multiple cues (irrespective of whether they are from the same modality or from different modalities), classify observations and perform hierarchical learning with them. Integration was achieved by simply concatenating the input vectors coming from different sources before feeding them to a GWR. To use a GWR network, which originally is an unsupervised learning method, as a classifier, the training algorithm was extended in two ways: The label provided alongside the input vector is either assigned to the newly created node or used to update the label of the best matching node during network adaptation. As for hierarchical learning, it was accomplished by serving the node activations of one GWR as input to the subsequent GWR in the hierarchy.

#### **Hyperparameter Optimization**

The research question was refined into a series of experiments where the goal of each was to find hyperparameter values that achieve the highest classification accuracy. The classifiers tested in such an experiment were either GWR networks trained on a subset of the considered object properties or neural classifiers implementing a particular integration strategy trained on the complete set of object properties. The experiments were carried out with *hyperopt* [6–8], a Python library for hyperparameter optimization. Table 2 is an overview of all the settings that were used in these hyperopt experiments. The *tree of Parzen estimators* (TPE) method was used for optimization and 500 trials were performed in each experiment.

**Table 2:** The settings used for the hyperopt experiments

General settings	
Number of trials	500
Optimization method	TPE
Configuration space	
Dist.	L2, L1, COS
*a*_*T*_, *h*_*T*_	[0.4, 0.8], [0, 0.2]
*𝜖*_*b*_, *𝜖*_*n*_	[0.2, 0.5], [0, 0.2]
*τ*_*b*_, *τ*_*n*_	[0.2, 0.5], [0, 0.2]
*π*, *α*_*T*_	200, [62.5, 250]

Considering too many dimensions in the configuration space might be problematic as 500 trials might not be enough to cover it appropriately, lowering the chances of finding good values. The ranges for most of the hyperparameters were therefore restricted to cover values that have been shown to work well in many instances (e.g. [44,51,52]). *π* was set to 200 to give the GWRs enough time to converge during training. As for Distance, three options were available, namely the *Euclidean distance* (or *L*_2_-*norm*, L2), the *Manhattan distance* (or *L*_1_-*norm*, L1) and the *cosine (dis)similarity* (COS). L2 loses its usefulness in high-dimensional space: Because the data becomes sparse in such a space, it becomes difficult to find best matching neurons for the input vectors. It has been shown that L1 is preferable to L2 for high-dimensional data [1]. COS does not take the vector magnitudes into account and hence could perform better than the other two options. The results of COS were used in degrees instead of radians as this matches the concept of distance better.

## Results

We set out to answer the research question of whether incorporating all three the brain-inspired processing principles in one integration strategy helps improve the classification accuracy. However, due to the complexity of the problem and systems used we need to answer a few additional questions beforehand:

1. How suitable are the contact microphones for tactile sensing? Which one of the four placements is the best to capture as much of the tactile vibrations as possible?
2. How discriminating is each object property? Are the object properties of one modality generally more discriminating than the other?
3. How discriminating are the unimodal and bimodal combinations of object properties as found in the first layer of the modality-based and brain-inspired integration strategies? Does combining object properties help?
4. Which of the three integration strategies yields the highest classification accuracy? Do they outperform the previous combinations?

The results that are featured in the following correspond to the (or a) best trial among all that were performed in the respective hyperopt experiment.

### **Using Contact Microphones as Tactile Sensors**

To assess the usefulness of the contact microphones as tactile sensors and to determine the optimal placement in the overall set-up for capturing discriminating tactile data, we classified the objects based on their tactile properties with the information coming from the four contact microphones shown in Fig. 5. For each tactile object property considered (i.e., texture and hardness) and for each of the four audio input channels, a separate hyperopt experiment was run. The results of their best trials are listed in Table 3.

**Table 3:** The suitability of the contact microphones as tactile sensors

The highlighted rows correspond to the hyperopt experiment, in which the highest classification accuracy was recorded

It is possible to discriminate objects based on the data from the contact microphones: In all best trials, the achieved classification accuracies were a lot higher than what would be achieved with random guessing (1/11 ≈ 9*%*). These results make the idea of attaching them to the robot from the inside, in particular the fingertips, worth considering.

With the inputs from the channel 0 microphone, which is the one that is the closest to where the tactile exploration of the objects takes place on the table, the highest accuracies were obtained for both texture and hardness. As this is the placement closest to the scene of exploration, it makes sense that the most discriminating tactile vibrations are captured here. The tactile data from this channel was hence used for the remaining experiments. Also, more observations were labeled correctly based on haptic texture than based on hardness for all channels. The overall results indicate that tactile sensing is indeed possible with the contact microphones.

### **Discriminating Power of the Object Properties**

Hyperopt experiments were performed for the visual and kinesthetic object properties as well to find out how discriminating these are and whether the object properties of one modality are more discriminating than the other ones. Table 4 shows for all object properties the trial that performed the best, also including the best trials for haptic texture and hardness for the sake of completeness.

**Table 4:** The discriminating power of the object properties.

The occurrence of L2 as the optimal distance function in the case of visual texture is an exception

In all the hyperopt experiments performed, there was only this one best trial with L2 as the distance function

The highlighted rows correspond to the hyperopt experiment, in which the highest classification accuracy was recorded

The most discriminating visual object property is color, while the least discriminating one is shape. Among the haptic object properties, shape is the most and hardness the least discriminating. No modality performed clearly better than the other at the level of these single object properties: Color and visual shape are also overall the most and least discriminating, respectively.

### **Uni-Versus Bimodal Combinations of Object Properties**

In the modality-based and brain-inspired integration strategies, the object properties are split up and processed in two separate streams in the first layer before their outputs are integrated in the next layer. Thus, it would be practical to quantify how well objects can be recognized based on each combination provided to one of these streams before turning towards the actual research question. It is especially interesting to know whether combining the object properties yields higher classification accuracies than when each object property is considered alone. That is why hyperopt experiments were conducted for these uni- and bimodal combinations, of which the best trials are summarized in Table 5.

**Table 5:** The discriminating power of some uni- and bimodal combinations of object properties

The highlighted rows correspond to the hyperopt experiment, in which the highest classification accuracy was recorded

More observations were classified correctly by combining the visual object properties than by combining the haptic ones. As for the bimodal combinations, a slightly higher classification accuracy was achieved with the combination of all material-related properties. Overall, the visual-only combination yields the best results. This is at the same time the only case where the classification based on a combination yields better results than any of the constituent object properties on its own. Judging by these results, it is likely that the modality-based integration strategy with the modality-specific processing streams performs better than the brain-inspired one, where the two streams are dedicated to processing the shape- and material-related object properties.

### **Performance of the Integration Strategies**

The final three hyperopt experiments were conducted to evaluate the integration strategies from Fig. 3 and to find out which one yields the best classification results. The best trials are shown in Table 6. To reduce the training time in the experiments for the modality-based and the brain-inspired integration strategies, the hyperparameters were optimized only for the second-layer components in charge of the actual classification. The hyperparameters for the first-layer components were taken from the previous experiments for the uni- and bimodal combinations of object properties (see Table 5). The information on the best trial provided in these two cases therefore only refer to this component in the second layer.

**Table 6:** The performance of the integration strategies

^a^ The provided information refer to the second-layer component

The highlighted rows correspond to the hyperopt experiment, in which the highest classification accuracy was recorded

As expected based on the previous results, the use of the modality-based integration strategy led to the best classification results with an accuracy of 86.4%, quite closely followed by the brain-inspired integration strategy with 81.8% and the monolithic integration strategy with 79.5%. While the full availability of the data (and not only portions of it as before) gives reason to expect higher classification accuracies than in the previous experiments, this is not the case for all integration strategies: Predicting the labels based on color or haptic shape alone results in a higher accuracy than when all the object properties are combined according to the monolithic integration strategy (see Table 4). Also, the number of correctly classified observations is higher when using only the visual object properties in combination than combining all object properties as in the modality-based integration strategy (see Table 5). In contrast, the use of the brain-inspired integration strategy improves the classification results compared to only considering the shape or the material properties (see Table 5).

## Discussion

The main goal of the hyperopt experiments presented in “Results” was to find out how well objects can be recognized with a visuo-haptic integration strategy that incorporates the brain-inspired processing principles from “Visuo-Haptic Principles of the Human Brain” as opposed to the integration strategies prevalent in the literature. In this setting, the use of the brain-inspired integration strategy did not lead to the highest performance, but we argue that its performance was still competitive.

A positive effect on the classification accuracy can definitely be observed for at least the first principle of hierarchical processing: The best classification accuracies achieved with the modality-based and the brain-inspired integration strategies, where the processing of the inputs is organized hierarchically and in two parallel streams, are higher than the one achieved with the monolithic integration strategy. As for the difference in the best accuracies obtained with these more complex integration strategies, it can be attributed to the different combinations of object properties are presented to these streams in each integration strategy: Less labels were predicted correctly with the brain-inspired integration strategy, where the shape and material-related inputs are processed separately (cf. the second principle), compared to the modality-based integration strategy, where the visual and haptic inputs are processed separately. Nevertheless, it is quite difficult to dismiss the second principle as not helpful based on this alone. A possible explanation could be issues with the quality of the data as hinted by the correlation between the optimal distance function and the 2-D projection of the input data.

**Fig. 7:**
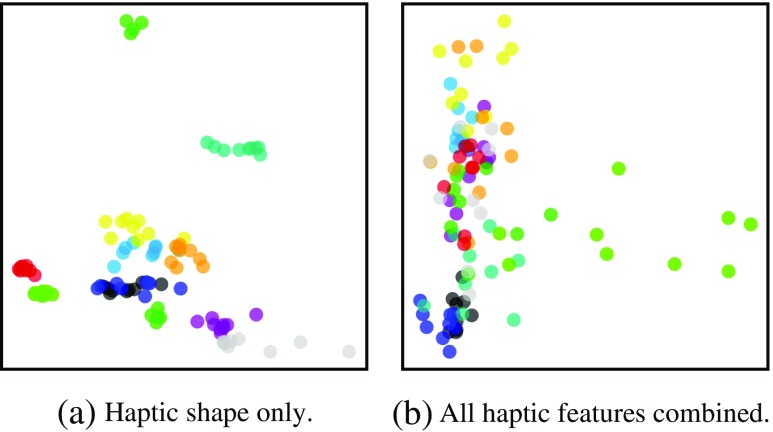
The 2-D projection of the input data for two of the hyperopt experiments (data points with the same color belong to the same object)

In all the hyperopt experiments that we ran, the optimal distance function turned out to be either L1 or COS, the only exception being the best trial for visual texture. This shows that L2 is usually not very useful in high dimensions (cf. [1]). We projected the input data (in its original dimensionality) for every hyperopt experiment onto 2-D space using PCA and looked at the distribution of the data points in the resulting scatter plot to find the following relation to the optimal distance function (see Fig. 7 for an example): When the data points belonging to the same object form more or less compact and distinguishable clusters, the distance function of the best trials in a hyperopt experiment is L1. COS appears as the optimal distance function when there is no real structure in how the data points are organized in this scatter plot. Mostly, the data points densely populate one particular area. For some objects, the data points are spread out quite widely. Considering the number of occurrence of COS as distance function in the best trials as well as the data dimensionalities in Table 4, the haptic object properties appear to be far more affected by the lack of structure than the visual ones. Also, the number of hyperopt experiments, where COS appears as distance function in the best trial, increases with the number of object properties considered as input as the lack of structure adds up in proportion to the dimensionality of each object property, see Tables 5 and 6. This explains why the best classification accuracy obtained with the brain-inspired integration strategy is slightly worse than the one obtained with the modality-based integration strategy: In the former, the information from the two modalities are integrated in the first layer already so that the visual data is affected earlier by the lack of structure in the haptic data than in the latter integration strategy.

COS increases the chances of finding some structure in the data by weakening the inductive bias of the learning algorithm: Originally, the assumption with the extended GWR is that neighbors in the feature space are likely to belong to the same class. COS projects the data points onto a unit hypersphere and measures neighborship on that hypersphere in terms of angles. Basically, the magnitude information is ignored and it is enough that data vectors point in the same direction. The fact that the inductive bias has to be modified in such a way to obtain a useful classifier in some of the experiments can be taken as an indication for the necessity of better data. Different hardware (i.e., a more dexterous robot equipped with better sensors, especially for tactile sensing) and a more sophisticated object exploration scheme might help in this respect. Especially, the result of the experiments with regard to the second principle of processing the shape and the material-related object properties in separate streams might change with better haptic data.

As for the effects of self-organization (cf. the third principle), they are difficult to assess because all three integration strategies were implemented using the same neural network model in an effort to make them easier to compare. In order to find out how advantageous incorporating this processing principle is to the classification accuracy, it would be good to compare for every integration strategy the performance of the brain-inspired implementation with that of other implementations not using any brain-inspired learning methods. Generally, future research with a larger dataset is needed to quantify how important each brain-inspired processing principle actually is for robot applications.

There are, of course, a few details with regard to how the parts of the brain responsible for performing visuo-haptic object recognition are organized in “Visuo-Haptic Principles of the Human Brain” that we omitted in our model for feasibility reasons. One such insight is that the sensory stimuli are processed hierarchically in the brain and increasingly abstract features are extracted in the process, see “Hierarchical Processing Along the Ventral Pathway for Object Identification” and “Haptic Activation Along the Substreams”. We extracted the features from the collected data using rather simple means. The *convolutional neural network* (CNN), for example, follows the principles of how visual stimuli are processed in the visual cortex and could therefore be examined in further investigations for the extraction of features in both modalities instead of the general-purpose feature extraction techniques used here. Another omitted detail is the difference in how the substreams along the ventral pathway for object shape and material processing themselves are organized (see “Substreams for Object Shape and Material Processing”). The LOtv is a bimodal convergence area, whereas the regions within the medial occipitotemporal cortex appear to be organized in crossmodally interacting foci that all process the information related to a particular material property. In the neural classifier that implements the brain-inspired strategy, both processing streams were simply modeled by instances of the self-organized GWR network. Finally, the brain is capable of combining the sensory information from the different modalities based on their reliability (see “Input-Driven Self-Organization of the Underlying Neural Circuits During Visuo-Haptic Integration”), which the self-organizing neural network model that was used in our implementations of the integration strategies to process the object-related information is not: The different types of information are integrated via vector concatenation so that the there is an implicit weighting of the object properties fixed by the length of the respective descriptors. When included, these details might actually prove to be helpful performance-wise.

## Conclusion

An increasing body of evidence suggests that multi- as well as crossmodal interactions between vision and haptics take place in the brain during object perception and recognition. This motivated us to take on the research question of whether the object recognition performance of artificial systems can be improved by incorporating the processing principles that are involved in the integration of the visual and haptic object-related information in the brain. Three such principles were derived here based on the current state of knowledge: the visual and haptic stimuli are processed hierarchically and in two separate streams, dedicated to object shape and material perception respectively, and the underlying mechanisms that perform the integration of the two modalities are self-organized.

To evaluate the importance of these three principles on the object recognition performance, an integration strategy that incorporates all of them was compared to the most common integration strategies in related works: A robot equipped with inexpensive contact microphones for tactile sensing was used to explore objects and extract various visual and haptic features from them. The different integration strategies were implemented as neural classifiers, which were then trained on the collected data in a series of hyperparameter selection experiments. Based on the results of these experiments, processing the visual and haptic inputs hierarchically and in two parallel streams seems to improve the object recognition performance, whereas organizing these streams to process the shape and material properties separately did not lead to the expected improvements.

In summary, our contributions are the following: 1. We identified three main processing principles that the visuo-haptic object recognition process in the brain is based on, 2. we considered a large number of haptic object properties in the evaluation of these principles and explored the concept of self-organization in the context of visuo-haptic object recognition, 3. we introduced the novel sensory concept of using inexpensive contact microphones to record vibrations across surfaces for tactile sensing and showed its feasibility, and 4. we found out that COS is good for measuring the distance between data points in high dimensions and should be preferred to L1 for the case of unstructured data.

## Footnotes

1 The full dataset is available online at https://figshare.com/s/555a20d9972e74fae355 https://figshare.com/s/555a20d9972e74fae355[63].
